# Influence of Critical Thinking Disposition, Clinical Reasoning Competence, and Nursing Practice Environment on Medication Safety Competence of Hospital Nurses

**DOI:** 10.3390/healthcare13050542

**Published:** 2025-03-03

**Authors:** Jeong An Oh, Eun A Kim, Hae Ran Kim

**Affiliations:** 1 Department of Nursing, Chonnam National University Hwasun Hospital, Gwangju 58128, Republic of Korea; wjddks1108@naver.com; 2 Department of Nursing, Honam University, Gwangju 62399, Republic of Korea; umbertoeo@hanmail.net; 3 Department of Nursing, College of Medicine, Chosun University, Gwangju 61452, Republic of Korea

**Keywords:** nurses, medication errors, safety, clinical competence, nursing environment, critical thinking

## Abstract

Objectives: This study examined the impact of nurses’ critical thinking disposition, clinical reasoning competency, and nursing practice environment on their medication safety competency. Methods: A cross-sectional design was employed, involving a convenience sample of 210 nurses from four tertiary general hospitals and two general hospitals. Data were collected in September 2023 utilizing structured online self-report questionnaires and analyzed utilizing descriptive statistics, independent *t*-tests, one-way analysis of variance, Pearson’s correlation coefficients, and stepwise multiple regression with SPSS/WIN 28.0 software. Results: Significant positive relationships (*p* < 0.001) were found between nurses’ medication safety competence and critical thinking disposition (r = 0.47), clinical reasoning competence (r = 0.67), and nursing practice environment (r = 0.40). Factors influencing medication safety competence were identified as clinical reasoning competence (β = 0.55, *p* < 0.001), nursing practice environment (β = 0.30, *p* < 0.001), and critical thinking disposition (β = 0.19, *p* < 0.001). The regression model accounted for 57% of the variance in medication safety competence and was statistically significant (F = 91.70, *p* < 0.001). Conclusions: These findings highlight key factors influencing medication safety competence of nurses and underscore the need for targeted strategies to enhance patient safety by optimizing critical thinking, clinical reasoning, and the nursing practice environment in healthcare settings.

## 1. Introduction

Unsafe medication practices and errors pose significant risks to patient safety, with the global economic burden of medication errors estimated at approximately USD 42 billion annually [1]. The World Health Organization (WHO) recognizes medication safety as a critical patient safety challenge [1]. In South Korea, despite the implementation of the Korean Patient Safety Reporting and Learning System—established to prevent patient safety incidents—the number of reported cases has continued to rise, from 9250 in 2018 to 14,820 in 2022. Among these incidents, 43.4% were attributed to medication errors [2]. The National Coordinating Council for Medication Error Reporting and Prevention (NCC MERP) defines medication errors as “any preventable event that may cause or lead to inappropriate medication use or patient harm while the medication is under the control of a healthcare professional, patient, or consumer” [3].

Medication errors can occur at various stages, including prescription, dispensation, and administration [2]. These errors not only compromise patient well-being but also prolong hospital stays and increase the risk of complications [4]. Nurses play a pivotal role in the medication administration process, serving as the final safeguard against errors and ensuring medication safety [5]. Therefore, nurses’ professional knowledge and advanced skills—collectively referred to as medication safety competencies—are essential in preventing and mitigating medication errors [6,7]. Medication safety competence encompasses the ability to accurately and safely perform tasks at each stage of the medication process, including patient-centered medication management, problem-solving, crisis management, interprofessional collaboration, and professional accountability. This competency is particularly crucial in healthcare systems such as South Korea’s, where nurses frequently face high patient turnover, complex clinical scenarios, and workforce shortages, making medication error prevention even more challenging [7,8,9].

Critical thinking is a fundamental skill in nursing practice [10] and has been widely recognized as a key factor influencing nurses’ medication safety competence [11]. The increasing complexity of the clinical environment, characterized by high patient acuity and rapid turnover rates, necessitates strong critical thinking skills among nurses [12]. Nurses with a high critical thinking disposition can enhance medication safety by adjusting dosages, anticipating potential adverse effects, and implementing preventive measures during the medication process [13,14].

Clinical reasoning is another essential competency for nurses who are decision-makers in clinical situations, as it involves integrating critical thinking with an understanding of a patient’s condition and clinical context [15]. Nurses with strong clinical reasoning skills are better equipped to assess health issues accurately, make informed decisions, and ultimately improve patient outcomes [16]. Consequently, clinical reasoning competence is considered a significant determinant of nurses’ medication safety competence [17].

The International Council of Nurses defines a positive nursing practice environment as one that supports professional practice [18], encompassing physical settings, interactions among team members, and organizational or institutional policies that influence nurses’ performance [19]. Nurses who perceive their work environment positively report lower burnout and turnover rates, contributing to improved care quality and patient safety [7,20]. A systematic review and meta-analysis identified high workloads, frequent night shifts, and nurse shortages as key factors contributing to increased medication errors within the practice environment [21,22].

In South Korea, where nurses often manage high patient acuity and demanding workloads, understanding how these factors collectively influence medication safety competence is essential for designing effective interventions. Recognizing the cultural and organizational nuances within South Korea’s healthcare system provides the opportunity to develop tailored educational programs and strategies to enhance patient safety outcomes [23].

In conclusion, critical thinking disposition, clinical reasoning competence and the nursing practice environment are essential factors influencing nurses’ medication safety competence. While global studies have examined the relationship between critical thinking disposition, clinical reasoning competence, and medication safety, research in the South Korean context remains limited. Investigating these variables within South Korean healthcare settings is essential for addressing context-specific challenges and bridging gaps in the existing literature [9,14]. This study aimed to address this gap by examining the effects of critical thinking disposition, clinical reasoning competence, and nursing practice environment on hospital nurses’ medication safety competence. The findings provide valuable insights for developing educational programs and strategies to enhance nursing practice environments and effectively manage factors influencing medication safety competence. The specific objectives of this study were:

1. To assess the levels and relationships among critical thinking disposition, clinical reasoning competence, nursing practice environment, and medication safety competence among hospital nurses.
2. To examine differences in critical thinking disposition, clinical reasoning competence, nursing practice environment, and medication safety competence based on hospital nurses’ characteristics.
3. To identify the factors influencing medication safety competence among hospital nurses.

## 2. Methods

### 2.1. Design and Participants

This study employed a descriptive survey design to examine the effects of critical thinking disposition, clinical reasoning competence, and the nursing practice environment on hospital nurses’ medication safety competence. Participants were conveniently sampled from six hospitals: four tertiary general hospitals and two general hospitals, each with more than 500 beds, across four regions (G City, S City, G Province, and J Province). Nurses were eligible to participate if they had at least three months of work experience, ensuring adequate exposure to clinical practice and direct involvement in medication-related nursing tasks. Participants had to understand the study’s purpose and voluntarily provide written informed consent. Nurses with less than three months of work experience were excluded, as they were in the orientation or adaptation period and could not be adequately assessed regarding their perceptions of the nursing practice environment or clinical judgment skills. Additionally, nurse managers were excluded due to the inclusion of leadership evaluation items in the nursing practice environment measurement tool.

The required sample size was calculated utilizing G*Power 3.1.9.4, with a significance level of ⍺ = 0.05, a power of 95%, and an effect size of 0.15, parameters selected to balance the statistical rigor and feasibility of data collection. With 14 predictors (three independent variables and 11 demographic characteristics), a minimum of 194 participants were needed. To account for an expected dropout rate of approximately 10%, data were collected from 210 participants. After excluding incomplete responses, data from 205 participants (97.6%) were included in the final analysis.

### 2.2. Measures

#### 2.2.1. Critical Thinking Disposition

Critical thinking disposition was assessed utilizing a validated South Korea tool [24] comprising 35 items across eight factors: intellectual integration (six items), creativity (four items), challenge (six items), openness (three items), prudence (four items), objectivity (four items), truth-seeking (three items), and inquisitiveness (five items). Nine items (items 3, 4, 9, 10, 14, 23, 26, 34, and 35) were reverse scored to enhance measurement accuracy. Responses were recorded on a 5-point Likert scale ranging from 1 (not at all true) to 5 (very true). The inclusion of reverse-scored items was carefully managed to minimize participant confusion and ensure accurate data interpretation, with higher scores indicating greater critical thinking disposition. In this study, the tool demonstrated strong internal consistency (Cronbach’s ⍺ = 0.91), with subscale reliability coefficients ranging from 0.70 to 0.87. 

#### 2.2.2. Clinical Reasoning Competence

Clinical reasoning competence was assessed utilizing the Korean version of the Nurse Clinical Reasoning Competence Scale [25,26]. This 15-item instrument employs a 5-point Likert scale ranging from 1 (not at all true) to 5 (very true), with higher scores reflecting greater clinical reasoning competence. The scale exhibited excellent internal consistency (Cronbach’s ⍺ = 0.94) in this study.

#### 2.2.3. Nursing Practice Environment

The nursing practice environment was evaluated utilizing a tool developed by South Korean clinical nurses [8], consisting of 20 items categorized into three subscales: managerial competence (six items), patient safety systems (eight items), and support systems (six items). Responses were rated on a 4-point Likert scale ranging from 1 (not at all true) to 4 (very true), with higher scores indicating more favorable perceptions of the nursing practice environment. The tool demonstrated strong internal consistency (Cronbach’s ⍺ = 0.93), with subscale reliability coefficients ranging from 0.83 to 0.94.

#### 2.2.4. Medication Safety Competence

Medication safety competence was assessed utilizing a South Korea developed instrument [9] comprising 36 items across six subscales: patient-centered medication management (nine items), problem solving (eight items), influencing factor management (six items), crisis management (six items), interprofessional collaboration (four items), and professional responsibility (three items). Responses were rated on a 5-point Likert scale ranging from 1 (not at all true) to 5 (very true), with higher scores indicating greater medication safety competence. The tool exhibited excellent reliability (Cronbach’s ⍺ = 0.97), with subscale reliability coefficients ranging from 0.72 to 0.92.

### 2.3. Procedure

To protect the participants’ rights, this study was conducted following approval from the Institutional Review Board of the researchers’ affiliated institutions (CNUHH-2023-167). Data were collected from 10 to 30 September 2023. Institutional leaders were informed of the study’s purpose and provided approval prior to the commencement of data collection. Eligible hospital nurses voluntarily participated in post reviewing the study description. The online survey, which took approximately 15 min to complete, was administered through Google Forms. To ensure anonymity, participants received unique codes through email to redeem gift cards as compensation. The consent form detailed the study’s benefits, risks of participation, confidentiality measures, voluntary withdrawal options, and the planned data destruction process three years post-study. To safeguard data integrity, survey links were distributed exclusively through institutional email addresses, restricting access to verified recipients and preventing unauthorized participation.

### 2.4. Statistical Analysis

Data were analyzed utilizing SPSS/WIN 28.0 (IBM Corp., Armonk, NY, USA). Descriptive statistics were utilized to summarize the participants’ characteristics and key variables (critical thinking disposition, clinical reasoning competence, nursing practice environment, and medication safety competence). Differences in these variables based on participant characteristics were analyzed utilizing independent *t*-tests and one-way analysis of variance (ANOVA), post checking for normality (skewness < [3] or kurtosis < [10]). Post hoc tests were conducted utilizing Scheffé’s test to analyze homogeneous variances, whereas Welch’s test and the Games-Howell test were employed for non-homogeneous variances to ensure robust statistical conclusions. Correlations among variables were analyzed utilizing Pearson’s correlation coefficients. Finally, stepwise multiple regression analysis identified the key factors influencing medication safety competence.

## 3. Results

### 3.1. General Characteristics

This study included 205 participants, most of whom were women (95.1%), with a mean age of 31.38 years. Most participants held a bachelor’s degree (88.3%) and were employed in tertiary general hospitals (75.1%), primarily in general wards (82.0%). The average length of work experience was 7.13 years, and 76.1% had received medication safety training within the past year. Additionally, 70.2% reported medication errors, including near-misses, while 54.1% reported medication safety incidents (Table 1).

### 3.2. Levels of Critical Thinking Disposition, Clinical Reasoning Competence, Nursing Practice Environment, and Medication Safety Competence

The mean scores for key variables were as follows: critical thinking disposition, 3.39 (±0.42); clinical reasoning competence, 3.75 (±0.50); nursing practice environment, 2.92 (±0.52); and medication safety competency, 3.98 (±0.53). Normality checks confirmed that all the variables followed a normal distribution (Table 2). Subscale scores exhibited that openness (3.79 ± 0.58) and influencing factors management (4.16 ± 0.56) had the highest scores among their respective categories, while creativity (2.92 ± 0.83) and support system (2.47 ± 0.67) had the lowest. Normality checks confirmed that all the variables followed a normal distribution (Table 2).

### 3.3. Correlations Among Critical Thinking Disposition, Clinical Reasoning Competence, Nursing Practice Environment, and Medication Safety Competence

Medication safety competence among hospital nurses was positively correlated with critical thinking disposition (r = 0.47, *p* < 0.001), clinical reasoning competence (r = 0.67, *p* < 0.001), and the nursing practice environment (r = 0.40, *p* < 0.001). Additionally, critical thinking disposition exhibited positive correlations with clinical reasoning competence (r = 0.44, *p* < 0.001) and the nursing practice environment (r = 0.15, *p* = 0.027). Clinical reasoning competence was also positively correlated with the nursing practice environment (r = 0.14, *p* = 0.048) (Table 3).

### 3.4. Factors Influencing Medication Safety Competence

A stepwise multiple regression analysis was conducted to identify the factors influencing hospital nurses’ medication safety competence. Critical thinking disposition, clinical reasoning competence, and the nursing practice environment emerged as significant predictors. Multicollinearity checks revealed tolerance values ranging from 0.80 to 0.97 (all exceeding 0.10) and variance inflation factors (VIFs) between 1.03 and 1.25 (all below 10), indicating no multicollinearity issues. The independence of errors was confirmed using a Durbin–Watson statistic of 1.94. The assumptions of normality and homoscedasticity were verified utilizing standardized residual analyses, including histograms, normal P-P plots, and scatterplots, all of which met the required criteria. No outliers were detected, as evidenced by Cook’s distance values of less than 1.0.

No significant differences in medication safety competence outcomes were observed according to the participants’ general characteristics. As a result, the regression analysis focused on three primary predictors: critical thinking disposition, clinical reasoning competence, and nursing practice environment.

The regression model identified clinical reasoning competence (β = 0.55, *p* < 0.001), nursing practice environment (β = 0.30, *p* < 0.001), and critical thinking disposition (β = 0.19, *p* < 0.001) as significant predictors of medication safety competence. The adjusted R^2^ value of the model was 0.57, indicating that the model could explain 57% of the variance in medication safety competence and that the model was statistically significant (F = 91.70, *p* < 0.001) (Table 4).

## 4. Discussion

This study aimed to identify the main factors influencing hospital nurses’ medication safety competence, with a focus on their critical thinking disposition, clinical reasoning competence, and the nursing practice environment. Additionally, this study explored the relationships among these variables and examined the differences based on participant characteristics, thereby addressing gaps in the existing literature.

Hospital nurses perceived their critical thinking disposition as moderately positive, with an average score of 3.39. This suggests their general ability to engage in reflective and evaluative thought processes in clinical scenarios. The results align with prior studies utilizing the same tool, reflecting specific characteristics and environmental differences between tertiary and large general hospitals [14,27]. Among the subdomains of critical thinking disposition, openness (3.79) had the highest score, whereas creativity (2.92) scored the lowest, consistent with prior findings. This discrepancy may be attributed to the nursing environment, which emphasizes rapid and accurate problem-solving and adherence to procedures as well as regulations [12,14]. Creativity is critical for generating new ideas, reorganizing existing information, and finding alternatives to problem-solving, particularly in dynamic and unpredictable clinical environments. However, the nature of nursing tasks often necessitates adherence to established procedures, which can limit the opportunities for creative thinking. Given the increasing importance of creativity in modern healthcare, there is a need for flexible organizational cultures and educational programs to nature creative thinking. For instance, innovative case-based learning and team-based problem-solving workshops could significantly enhance nurses’ creativity [12,27].

Clinical reasoning competence was perceived as relatively high, with an average score of 3.75, which is consistent with prior studies and underscores its importance in accurately assessing patient conditions and ensuring medication safety. However, differences were observed depending on participant characteristics and data collection methods [15,25]. Notably, studies measuring clinical reasoning competence among clinical nurses remain limited, and results should be interpreted with caution due to variations in sample size and data collection methods. While simulation and case-based teaching methods are increasingly utilized in nursing education to improve clinical reasoning competence among students, there is a lack of targeted studies and programs aimed at clinical nurses [28,29]. Continued development and expansion of educational programs are essential for enhancing clinical reasoning competence in clinical settings. This finding directly supports the first objective of this study, which was to explore the relationship between critical thinking disposition, clinical reasoning competence, and medication safety competence.

Hospital nurses perceived their nursing practice environment as moderately positive, with an average score of 2.92. This score was slightly higher than those reported in prior studies, suggesting that institutional support and resources may have contributed to this perception. The discrepancy between this study and prior studies may stem from differences in the tools utilized and participant characteristics [7,8]. The utilization of a recently developed tool for South Korean nurses, along with the inclusion of tertiary and large general hospitals, underscores the significance of this study. However, differences in prior studies that utilized adapted tools and specific nursing roles underscore the need for careful examination of tool components and participant selection criteria in future studies to enhance reliability and validity.

Medication safety competence was relatively high, with an average score of 3.98, consistent with prior studies [9], reflecting the effectiveness of standardized protocols and training in maintaining safety standards. No significant differences in medication safety competencies were observed based on hospital type, possibly as a result of the standardized working environments and policies across institutions [17]. This finding addresses the second objective of the study, which was to examine differences in medication safety competence based on participant characteristics. However, this study faced limitations in balancing the proportion of hospital types as a result of recruitment challenges, suggesting that future research should ensure more balanced sampling across hospital types [23]. Such efforts could contribute to the development of systematic and targeted strategies to improve nurses’ medication safety competence [7].

This study identified critical thinking disposition, clinical reasoning competence, and nursing practice environment as major predictors of hospital nurses’ medication safety competence, with clinical reasoning competence (β = 0.55), nursing practice environment (β = 0.30), and critical thinking disposition (β = 0.19) being significant factors. These findings highlight the importance of clinical reasoning in comprehensively assessing patient conditions and safely administering medications in accordance with hospital protocols and guidelines. Consistent with prior studies [9,23], this study confirmed that both clinical reasoning competence and the nursing practice environment significantly impact medication safety competence. In contrast to prior research conducted predominantly in Western settings, this study provides a unique contribution by examining these factors within the Korean healthcare system, highlighting cultural and organizational differences [7]. Prior research supports a close relationship between critical thinking disposition and clinical reasoning competence, which are both pivotal in preventing medication errors and promoting patient safety [4,9,10,20]. This study therefore directly addresses the third objective of identifying major factors influencing nurses’ medication safety competence. To enhance clinical reasoning competence, diverse education and training programs are necessary, as it is challenging to improve this skill over a short period. Simulation-based training and case-based video education have proven effective in strengthening clinical reasoning competence [28,29]. Furthermore, collecting medication error cases from clinical settings and providing case-based education, both online and offline, could further enhance nurses’ clinical reasoning skills [5,7].

The nursing practice environment has emerged as another critical factor, with organizational settings playing a significant role in influencing nurses’ medication safety competence [9,28]. Studies have shown that improved communication, adequate staffing, and robust medication policies are essential for preventing medication errors [5,7]. Environmental factors such as communication satisfaction and safety culture also play pivotal roles in promoting medication safety [8,14]. These findings underscore the importance of organizational strategies that support and manage nursing practice environments. Efforts to foster a positive nursing practice environment, through structured policy application, resource management, and systematic development human resource development, are essential for fostering safe and effective nursing practices [30]. Positive changes in the nursing practice environment can improve nursing performance and reduce patient safety. While this study focused primarily on medication safety competencies within the Korean nursing context, similar challenges have been reported globally. For instance, the event reports used by the UK’s NHS and the US’s PS emphasize the significant impact of nursing practice environments and clinical reasoning competence in ensuring medication safety. These findings align with the current study’s findings, suggesting that enhancing nurses’ critical thinking and clinical environments can be universally beneficial in reducing medication errors. Future studies could expand on this work by including data from diverse healthcare systems to provide a more comprehensive understanding of global trends in medication safety [1,3,4].

Critical thinking disposition was also identified as a significant predictor of medication safety competence [3,9,10,11]. Nurses with strong critical-thinking skills are better equipped to anticipate and mitigate risks during medication administration, ensuring safe patient care [16,17]. Some prior studies have focused on nurses in tertiary and general hospitals within specific regions, necessitating caution when generalizing their findings. Future studies should aim for broader representation by including diverse healthcare institutions and regions to validate these findings.

This study has several strengths, including its focus on a largely underexplored population, hospital nurses in South Korea, and the use of validated instruments. Prior studies have predominantly focused on Western contexts, and there is limited research on the Korean nursing population, making this study a unique contribution [23]. However, this study also has limitations, such as the reliance on self-reported data, which may introduce response bias. Additionally, challenges in achieving a balanced sample across hospital types could impact the findings. Self-reported data may lead to overestimation or underestimation of competencies due to social desirability or recall bias [14]. Future research should address these limitations by incorporating a more diverse participant pool and utilizing objective data collection methods. For instance, observational or performance-based assessments alongside self-reports may provide a more comprehensive understanding of medication safety competence [9,14].

Based on these findings, we recommend that healthcare institutions implement targeted training programs to improve nurses’ critical thinking disposition and clinical reasoning competencies. Simulation-based education and case-based learning have proven effective in enhancing clinical reasoning and decision-making skills [28]. Moreover, improving nursing practice environments through organizational support, adequate staffing, and robust medication policies is crucial for fostering medication safety competence. The evidence suggests that enhancing communication satisfaction, reducing workload, and providing sufficient resources can significantly enhance nursing performance and patient safety [7,23]. Furthermore, integrating medication safety policies and providing regular training on error prevention can further support safe nursing practices [9].

## 5. Conclusions

This study identified critical thinking disposition, clinical reasoning competence, and the nursing practice environment as key factors influencing hospital nurses’ medication safety competence. Among these, clinical reasoning competence (β = 0.55) emerged as the strongest predictor, emphasizing its crucial role in patient assessment and safe medication administration. To enhance this skill, simulation-based training and case discussions are recommended. The nursing practice environment (β = 0.30) also played a significant role, emphasizing the importance of organizational support. Strengthening communication, ensuring adequate staffing, and fostering a culture of safety are essential steps in improving medication safety. Additionally, critical thinking disposition (β = 0.19) was found to be relevant in anticipating risks and ensuring safe medication practices, suggesting that reflection and problem-solving exercises can help nurture competence. This study effectively addressed its first objective by demonstrating significant positive relationships between critical thinking disposition, clinical reasoning competence, nursing practice environment, and medication safety competence. It also fulfilled the second objective by showing no significant differences in medication safety competence based on hospital type. However, the findings underscore the need for a balanced sample distribution across hospital types in future studies. Overall, the findings suggest that targeted education and improvements in the work environment can enhance nurses’ medication safety competence and contribute to improved patient outcomes. To address these findings, healthcare institutions should integrate structured training programs focused on simulation-based and case-based learning into their professional development initiatives while fostering a supportive nursing practice environment.

Future research should expand the study sample to include diverse healthcare institutions to improve the generalizability of the findings. Longitudinal studies are needed to evaluate the sustained effects of training programs on medication safety competency. Further investigation of the interaction between organizational culture and individual competencies is essential to understanding how they collectively contribute to the promotion of safe medication practices. Research that integrates organizational improvements with competency development may offer valuable insights into achieving a sustainable reduction in medication errors.

## Figures and Tables

**Table 1:** Differences in Medication Safety Competence by Participant Characteristics (*N* = 205).

Characteristics	Categories	*N* (%)	Medication Safety Competence	
			M ± SD	t or F (*p*)
Sex	Male	10 (4.9)	4.11 ± 0.61	0.80 (0.425)
	Female	195 (95.1)	3.97 ± 0.52	
Age (years)	≤29	92 (44.9)	3.96 ± 0.55	1.74 (0.178)
(M ± SD: 31.38 ± 5.66)	30~39	87 (42.4)	3.94 ± 0.52	
	≥40	26 (12.7)	4.15 ± 0.46	
Marital status	Single	144 (70.2)	3.94 ± 0.53	−1.71 (0.088)
	Married	61 (29.8)	4.07 ± 0.50	
Education	3-year diploma	6 (2.9)	3.85 ± 0.77	0.62 (0.542)
	4-year bachelor	18 1 (88.3)	3.97 ± 0.52	
	≥Master	18 (8.8)	4.09 ± 0.50	
Hospital type	Tertiary Hospital	154 (75.1)	3.97 ± 0.52	−0.23 (0.821)
	General hospital	51 (24.9)	3.99 ± 0.56	
Department	Ward	168 (82.0)	3.98 ± 0.54	0.33 (0.740)
	ICU·ER *	37 (18.0)	3.95 ± 0.47	
Total career duration (years)	<1	15 (7.3)	3.68 ± 0.62	1.82 (0.145)
(M ± SD: 7.13 ± 5.62)	1–<5	70 (34.1)	3.99 ± 0.52	
	5–<10	68 (33.2)	4.02 ± 0.50	
	≥10	52 (25.4)	3.98 ± 0.53	
Career in present	<1	60 (29.3)	3.88 ± 0.61	1.00 (0.396)
work unit (year)	1–<3	79 (38.5)	4.02 ± 0.49	
(M ± SD: 2.50 ± 2.63)	3–<5	38 (18.5)	3.98 ± 0.50	
	≥5	28 (13.7)	4.05 ± 0.45	
Medication safety education (within the last year)	Yes	156 (76.1)	3.99 ± 0.55	0.50 (0.616)
	No	49 (23.9)	3.94 ± 0.46	
Experience of medication errors (including near misses)	Yes	143 (70.2)	3.98 ± 0.51	0.13 (0.899)
	No	62 (29.8)	3.97 ± 0.56	
Experience of reporting medication safety accidents (including near misses)	Yes	111 (54.1)	4.03 ± 0.48	1.52 (0.129)
	No	94 (45.9)	3.92 ± 0.57	

M = Mean; SD = Standard deviation; * ICU·ER = intensive care unit·emergency room.

**Table 2:** Levels of Critical Thinking Disposition, Clinical Reasoning Competence, Nursing Practice Environment, and Medication Safety Competence (*N* = 205).

Variables/Sub-Categories		Possible Range	Item Range	Skewness	Kurtosis	M ± SD
Critical thinking disposition		1–5	2.26–4.77	0.09	0.18	3.39 ± 0.42
	Intellectual integrity	1–5	2.17–5.00	−0.34	0.58	3.74 ± 0.48
	Creativity	1–5	1.00–5.00	0.04	−0.43	2.92 ± 0.83
	Challenge	1–5	1.67–4.67	0.00	−0.50	3.05 ± 0.60
	Open-mindedness	1–5	1.67–5.00	−0.37	0.88	3.79 ± 0.58
	Prudence	1–5	1.50–5.00	0.08	−0.16	3.40 ± 0.62
	Objectivity	1–5	1.00–5.00	−0.36	0.35	3.25 ± 0.65
	Trust seeking	1–5	1.00–5.00	−0.55	1.11	3.54 ± 0.64
	Inquisitiveness	1–5	1.00–5.00	−0.57	0.59	3.50 ± 0.64
Clinical reasoning competence		1–5	2.00–5.00	−0.15	0.79	3.75 ± 0.50
Nursing practice environment		1–4	1.35–4.00	−0.22	0.09	2.92 ± 0.52
	Safety management system of nurse	1–4	1.63–4.00	−0.59	0.24	3.16 ± 0.56
	Competence of nursing manager	1–4	1.00–4.00	−0.26	−0.58	3.03 ± 0.69
	Support system of nurse	1–4	1.00–4.00	0.16	−0.26	2.47 ± 0.67
Medication safety competence		1–5	2.69–5.00	0.15	−0.33	3.98 ± 0.53
	Patient-centered medication management	1–5	2.67–5.00	−0.01	−0.31	4.05 ± 0.55
	Improvement of problem situation	1–5	2.00–5.00	0.02	−0.20	3.84 ± 0.61
	Management of influence factors	1–5	2.50–5.00	−0.29	−0.18	4.16 ± 0.56
	Crisis management	1–5	2.67–5.00	−0.05	−0.65	4.07 ± 0.61
	Interdisciplinary collaboration	1–5	1.00–5.00	−0.43	0.14	3.71 ± 0.82
	Responsibility as a nursing professional	1–5	2.33–5.00	−0.07	−0.58	3.90 ± 0.69

M = Mean; SD = Standard deviation.

**Table 3:** Correlations Among Critical Thinking Disposition, Clinical Reasoning Competence, Nursing Practice Environment, and Medication Safety Competence (*N* = 205).

Variables	Critical Thinking Disposition	Clinical Reasoning Competence	Nursing Practice Environment
	r (*p*)	r (*p*)	r (*p*)
Clinical reasoning competence	0.44 (<0.001)		
Nursing practice environment	0.15 (0.027)	0.14 (0.048)	
Medication safety competence	0.47 (<0.001)	0.67 (<0.001)	0.40 (<0.001)

**Table 4:** Factors Influencing Medication Safety Competence (*N* = 205).

Variables	B	SE	β	t	*p*
(Constant)	0.14	0.24		0.59	0.556
Clinical reasoning competence	0.58	0.05	0.55	10.76	<0.001
Nursing work environment	0.30	0.05	0.30	6.35	<0.001
Critical thinking disposition	0.23	0.06	0.19	3.65	<0.001
R^2^ = 0.58, Adj.R^2^ = 0.57, F = 91.70, *p* < 0.001, Durbin–Watson = 1.94					

B = Unstandardized coefficient; β = Standardized coefficient; SE = Standard error; Adj = Adjusted.

## Data Availability

The datasets analyzed in this study are available from the corresponding author upon reasonable request as a result of privacy concerns.

## References

[1] World Health Organization (2017). Medication Without Harm-Global Patient Safety Challenge on Medication Safety. https://apps.who.int/iris/bitstream/handle/10665/255263/WHO-HIS-SDS-2017.6-eng.pdf?sequence=1.

[2] Korea Institute for Healthcare Accreditation (2022). Korean Patient Safety Incident Report 2022. https://www.kops.or.kr/portal/board/statAnlrpt/boardList.do.

[3] (2024). National Coordinating Council for Medication Error Reporting and Prevention. Consumer Information for Safe Medication Use. https://www.nccmerp.org/consumer-information.

[4] Alrabadi N., Shawagfeh S., Haddad R., Mukattash T., Abuhammad S., Al-Rabadi D., Abu Farha R., AlRabadi S., Al-Faouri I. (2021). Medication errors: A focus on nursing practice. J. Pharm. Health Serv. Res..

[5] Tang F.I., Sheu S.J., Yu S., Wei I.L., Chen C.H. (2007). Nurses relate the contributing factors involved in medication errors. J. Clin. Nurs..

[6] Kavanagh C. (2017). Medication governance: Preventing errors and promoting patient safety. Br. J. Nurs..

[7] Wan Q., Li Z., Zhou W., Shang S. (2018). Effects of work environment and job characteristics on the turnover intention of experienced nurses: The mediating role of work engagement. J. Adv. Nurs..

[8] Ko Y.J., Hong G.R.S. (2022). Development and evaluation of nursing work environment scale of clinical nurses. J. Korean Acad. Nurs. Adm..

[9] Park J., Seomun G. (2021). Development and validation of the medication safety competence scale for nurses. West. J. Nurs. Res..

[10] Christianson K.L. (2020). Emotional intelligence and critical thinking in nursing students: Integrative review of literature. Nurse Educ..

[11] Martyn J.A., Paliadelis P., Perry C. (2019). The safe administration of medication: Nursing behaviours beyond the five-rights. Nurse Educ. Pract..

[12] Lee D.S., Abdullah K.L., Subramanian P., Bachmann R.T., Ong S.L. (2017). An integrated review of the correlation between critical thinking ability and clinical decision-making in nursing. J. Clin. Nurs..

[13] Jones J.R., Boltz M., Allen R., Van Haitsma K., Leslie D. (2022). Nursing students’ risk perceptions related to medication administration error: A qualitative study. Nurse Educ. Pract..

[14] Sun Y., Yin Y., Wang J., Ding Z., Wang D., Zhang Y., Zhang J., Wang Y. (2023). Critical thinking abilities among newly graduated nurses: A cross-sectional survey study in China. Nurs. Open.

[15] Bae J., Lee J., Choi M., Jang Y., Park C.G., Lee Y.J. (2023). Development of the clinical reasoning competency scale for nurses. BMC Nurs..

[16] Griffits S., Hines S., Moloney C. (2023). Characteristics and processes of registered nurses’ clinical reasoning and factors relating to the use of clinical reasoning in practice: A scoping review. JBI Evid. Synth..

[17] Rohde E., Domm E. (2018). Nurses’ clinical reasoning practices that support safe medication administration: An integrative review of the literature. J. Clin. Nurs..

[18] Woo B.F.Y., Lee J.X.Y., Tam W.W.S. (2017). The impact of the advanced practice nursing role on quality of care, clinical outcomes, patient satisfaction, and cost in the emergency and critical care settings: A systematic review. Hum. Resour. Health.

[19] Déry J., Paquet M., Boyer L., Dubois S., Lavigne G., Lavoie-Tremblay M. (2022). Optimizing nurses’ enacted scope of practice to its full potential as an integrated strategy for the continuous improvement of clinical performance: A multicentre descriptive analysis. J. Nurs. Manag..

[20] Al-Hamdan Z., Manojlovich M., Tanima B. (2017). Jordanian nursing work environments, intent to stay, and job satisfaction. J. Nurs. Scholarsh..

[21] Fathizadeh H., Mousavi S.S., Gharibi Z., Rezaeipour H., Biojmajd A.-R. (2024). Prevalence of medication errors and its related factors in Iranian nurses: An updated systematic review and meta-analysis. BMC Nurs..

[22] Sabzi Z., Mohammadi R., Talebi R., Roshandel G.R. (2019). Medication errors and their relationship with care complexity and work dynamics. Open Access Maced. J. Med. Sci..

[23] Kwon I.S., Lee G.E., Kim G.D., Kim Y.H., Park K.M., Park H.S., Sohn S.K., Lee W.S., Jang K.S., Chung B.Y. (2006). Development of a critical thinking disposition scale for nursing students. J. Korean Acad. Nurs..

[24] Alshehri F.D., Jones S., Harrison D. (2023). The effectiveness of high-fidelity simulation on undergraduate nursing students’ clinical reasoning-related skills: A systematic review. Nurse Educ. Today.

[25] Liou S.R., Liu H.C., Tsai H.M., Tsai Y., Lin Y., Chang C., Cheng C. (2016). The development and psychometric testing of a theory-based instrument to evaluate nurses’ perception of clinical reasoning competence. J. Adv. Nurs..

[26] Joung J., Han J.W. (2017). Validity and reliability of a Korean version of nurse clinical reasoning competence scale. J. Korea Acad.-Ind. Coop. Soc..

[27] Kim K.Y., Lee E. (2016). The relationship among critical thinking disposition, nursing process competency and evidence-based practice competency in nurses working in hospitals. J. Korean Data Inf. Sci. Soc..

[28] Kim J.H., Kim S.H., Park E.Y., Kwon I.G. (2021). Development and application of case-based video education for oncology nursing based on clinical reasoning. Asian Oncol. Nurs..

[29] Lake E.T., Sanders J., Duan R., Riman K.A., Schoenauer K.M., Chen Y. (2019). A meta-analysis of the associations between the nurse work environment in hospitals and 4 sets of outcomes. Med. Care.

[30] Arkin L., Schuermann A.A., Loerzel V., Penoyer D. (2023). Exploring medication safety practices from the nurse’s perspective. Am. J. Nurs..

